# Genetic diversity in European *Pisum* germplasm collections

**DOI:** 10.1007/s00122-012-1839-1

**Published:** 2012-04-01

**Authors:** R. Jing, M. A. Ambrose, M. R. Knox, P. Smykal, M. Hybl, Á. Ramos, C. Caminero, J. Burstin, G. Duc, L. J. M. van Soest, W. K. Święcicki, M. G. Pereira, M. Vishnyakova, G. F. Davenport, A. J. Flavell, T. H. N. Ellis

**Affiliations:** 1 Division of Plant Sciences, University of Dundee at JHI, Invergowrie, Dundee, DD2 5DA UK; 2 John Innes Centre, Norwich Research Park, Colney Lane, Norwich, NR4 7UH UK; 3 Agritec Plant Research Ltd., Zemedelska 2520/16, 787 01 Sumperk, Czech Republic; 4 Department of Botany, Faculty of Sciences, Palacký University, Slechtitelu 11, 783 71 Olomouc, Czech Republic; 5 Centro para la calidad de los alimentos, INIA, Campus universitario, 42004 Soria, Spain; 6 Instituto Tecnológico Agrario, Consejería de Agricultura y Ganadería de la Junta de Castilla y León, Ctra Burgos, km 119, 47071 Valladolid, Spain; 7 Institut National de la Recherche Agronomique (INRA), UMR LEG, 17 rue de Sully-Building B1, Office 110, BP 86510, 21065 Dijon Cédex, France; 8 Centre for Genetic Resources, The Netherlands (CGN), P. O. Box 16, 6700 AA Wageningen, The Netherlands; 9 Institute of Plant Genetics, Polish Academy of Sciences, ul. Strzeszyńska 34, 60-479 Poznan, Poland; 10 Instituto Nacional de Investigação Agrária, Apartado 6, 7350-951 Elvas, Portugal; 11 N.I. Vavilov Institute of Plant Industry (VIR), Bolshaya Morskaya Street 42-44, 190000 St. Petersburg, Russian Federation; 12 Crop Informatics, 211 Malecon Armenariz, Miraflores, Lima, Peru; 13 Institute of Biological, Environmental and Rural Sciences, Aberystwyth University, Gogerddan, Aberystwyth, SY23 3EB UK; 14 Present Address: Institut für Biochemie und Biologie, Universität Potsdam, Karl-Liebknecht-Str. 24-25, Haus 26, 14476 Potsdam-Golm, Germany

## Abstract

The distinctness of, and overlap between, pea genotypes held in several *Pisum* germplasm collections has been used to determine their relatedness and to test previous ideas about the genetic diversity of *Pisum.* Our characterisation of genetic diversity among 4,538 *Pisum* accessions held in 7 European Genebanks has identified sources of novel genetic variation, and both reinforces and refines previous interpretations of the overall structure of genetic diversity in *Pisum.* Molecular marker analysis was based upon the presence/absence of polymorphism of retrotransposon insertions scored by a high-throughput microarray and SSAP approaches. We conclude that the diversity of *Pisum* constitutes a broad continuum, with graded differentiation into sub-populations which display various degrees of distinctness. The most distinct genetic groups correspond to the named taxa while the cultivars and landraces of *Pisum sativum* can be divided into two broad types, one of which is strongly enriched for modern cultivars. The addition of germplasm sets from six European Genebanks, chosen to represent high diversity, to a single collection previously studied with these markers resulted in modest additions to the overall diversity observed, suggesting that the great majority of the total genetic diversity collected for the *Pisum* genus has now been described. Two interesting sources of novel genetic variation have been identified. Finally, we have proposed reference sets of core accessions with a range of sample sizes to represent *Pisum* diversity for the future study and exploitation by researchers and breeders.

**Electronic supplementary material:**

The online version of this article (doi:10.1007/s00122-012-1839-1) contains supplementary material, which is available to authorized users.

## Introduction

The diversity and taxonomy of *Pisum* has received considerable attention (Ellis et al. 1998; Pearce et al. 2000; Simioniuc et al. 2002; Vershinin et al. 2003; Baranger et al. 2004; Coyne et al. 2005; Tar’an et al. 2005; Espósito et al. 2007; Smýkal et al. 2008; Zong et al. 2009; Jing et al. 2010, Martin-Sanz et al. 2011; reviewed in Ellis 2011 and Smýkal et al. 2011). A consensus has emerged from these studies that the genus as a whole represents a broad continuum comprising two wild species *Pisum elatius* and *Pisum fulvum* (Vershinin et al. 2003) together with distinct domesticated groups or taxa, such as *Pisum abyssinicum* and “cv. Afghanistan” (Westphal 1974; Young and Matthews 1982; Jing et al. 2010). The wild form, *P. elatius*, is remarkably diverse and almost as broad as the genus as a whole. *P. elatius* has also been taken to include other named groups that are not monophyletic or comprise taxa that are no more distinct than other *P. elatius* accessions (Vershinin et al. 2003; Jing et al. 2005; Ellis 2011).

A notable exception to this broad consensus was the description of an extensive collection of *Pisum* accessions focussed on diverse Chinese material (Zong et al. 2008, 2009).This has been discussed in relation to other germplasm (Smýkal et al. 2011) and while it appears that the Chinese material is indeed diverse (consistent with Jing et al. 2010), some features of the data such as the fragmentation proposed for *P. fulvum* does not seem to be well supported. A possible explanation for this fragmentation may be homoplasy, and this was discussed in relation to the relative mutation rates of different marker types by Ellis (2011).

The structure of genetic diversity in the John Innes *Pisum* germplasm collection has been described recently (Jing et al. 2010). This germplasm collection, which contains 1,200 *Pisum sativum* cultivars, 600 traditional landraces and 750 wild *Pisum* samples, together with genetic stocks and reference lines from other collections, is the most complete assembly of *Pisum* germplasm to be studied to date by marker analysis. A subset of the previously studied accessions that had been strongly assigned to STRUCTURE sub-groups by Jing et al. (2010) have been genotyped at 1,484 SSAP defined loci, while the new accessions were genotyped using 27 retrotransposon-based insertion polymorphisms (RBIPs) scored in high throughput by the tagged microarray marker (TAM) microarray method (Flavell et al. 2003). RBIPs are based on the insertions of LTR retrotransposons (mainly *PDR1*; Jing et al. 2005) and the use of PCR-based detection of the presence and/or the absence of single retrotransposon insertions by combining two primers flanking the insertion site with a single outward-priming transposon-specific primer (Flavell et al. 1998). Thus, RBIP yields codominant marker scores for the irreversible sequence differences, which are well suited to studying diversity at the genus level (Jing et al. 2010). The polymorphism data for the JI *Pisum* collection were analysed using both the program STRUCTURE (Pritchard et al. 2000) and multifactorial approaches (Perrier et al. 2003). The former approach yielded a stratified description of genetic diversity that comprised three primary STRUCTURE groups (groups 1–3) corresponding roughly to landrace, cultivar and wild samples, respectively. Sequential STRUCTURE analysis of these groups revealed sub-structuring into 14 sub-groups, many of which correlated well with the taxonomic sub-divisions, domestication-related traits and/or geographical distributions for the corresponding samples.

Here, we extend the analysis of *Pisum* diversity by including a further 1,518 *Pisum* accessions selected from 6 other major European collections (Table 1). The main objectives of this study were to determine whether our earlier broad conclusions for the genetic structure of *Pisum* is supported by adding germplasm from across Europe, to determine the extent of distinctness of germplasm held in different germplasm centres and to propose a representative set of *Pisum* accessions for the future study and exploitation.

**Table 1:** Participating European collections and details of the material analysed

Genebank	FAO code	Number of accessions and focus of selection
ITACyL, Instituto Tecnológico Agrario de Castilla y León, Valladolid, Spain	ESP109	347 comprising 270 Spanish landrace accession with sub-accessions making up the balance
INRA, Station de Génétique et d’Amélioration des Plantes, Dijon, France	FRA043	360 representing the French core collection with external reference lines
CGN, Centre for Genetic Resources, the Netherlands, Wageningen, NLD	NLD037	172 are landrace accessions originating from Asiatic highlands
Poznanska Hodowla Roslin, Plant Breeding Station, Wiatrowo, Poland, POL 004	POL003	364 broad selection including wild accessions, landraces, cultivars and genetic stocks
EAN-BANCO, Banco de Germoplasma, Genetica Estacao Agronomica Nacional, Oeiras, Portugal	PRT005	52 Portuguese landraces accessions
N.I. Vavilov Research Institute of Plant Industry (VIR), St. Petersburg, Russia	RUS001	305 cultivated forms including 116 from across Russia

## Materials and methods

### Plant material

We analysed the 3,020 John Innes *Pisum* germplasm accessions described in (Jing et al. 2010), plus 9 duplicates and 1,518 accessions from 6 other European germplasm collections (listed in Table 1). Access to this extended germplasm was facilitated through the Working Group for Grain Legumes of the European Cooperative Programme for Crop Genetic Resources (ECPGR). The complete list of accessions is in Supplementary Table 1.

### Plant growth and DNA preparation

Single plants for each accession were grown at each of the six germplasm centres, then dried leaf segments stored in silica gel were sent to a single location (Scotland) for DNA extraction by the Qiagen DNeasy 96 method.

### RBIP markers and TAM microarray-based marker analysis

27 RBIP markers were selected from an original set of 45 (Jing et al. 2010) on the basis of their informativeness (allele frequencies in the JI collection), reliability and data quality (signal to noise ratio, Cy3/Cy5 ratio). Marker scoring was as described in Jing et al. (2010), with the addition of a dye swap (hybridization of Cy3-labelled and Cy5-labelled probes to separately arrayed aliquots from the same PCR reaction set).

### SSAP markers

*PDR1* SSAPs are amplicons derived from *Taq*I digested genomic DNAs to which adaptors have been ligated. The PCR uses two base selective primers corresponding to the adaptor and a labelled retrotransposon primer directed towards the 3′ 156 bp LTR that lacks *Taq*I restriction sites (Ellis et al. 1998). In the present study, these were screened as fluorescent markers on an ABI 3730 xl platform using 16 selective *PDR1* primer combinations carrying all possible 2-base 3′ extensions on the adaptor primer (Knox et al. 2009).

### Genetic diversity data analysis

Genotypic scores were collated and analysed as described in Jing et al. (2010), with the Dice genetic distance measures used in this analysis calculated using DARwin5 (Perrier et al. 2003). The program STRUCTURE (Pritchard et al. 2000; Pritchard and Wen 2004) and the method of Evanno et al. (2005) were used to model potential relationships between accessions. Correlation analysis between STRUCTURE runs (Supplementary Figure 1) was performed as follows. 

For each run (*r*), each accession has a value of *Q*_rp_ corresponding to the presumed contribution of each proposed progenitor population (*p*). The correlation between pairs of runs was calculated using Genstat v13 (Payne et al. 2008) as the average of the absolute value of all possible pairwise correlations of populations. Note that not all runs were equally well self-correlated as this depends on the correlation between populations within a run.

**Fig. 1:**
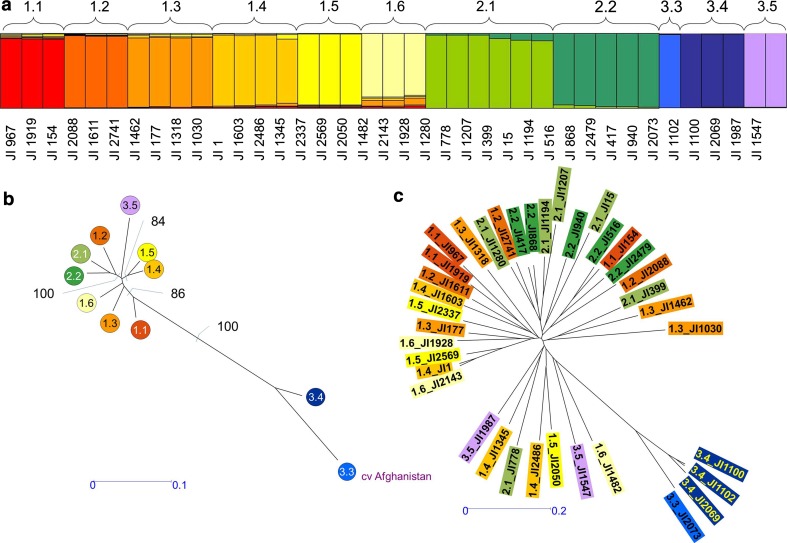
SSAP analysis of strongly assigned accessions. **a** Assignment of *Q* from Jing et al*.* (2010). **b** Neighbour joining (NJ) tree calculated using DARwin5 (Perrier et al*.* 2003). Allele frequency within the population sub-groups of Jing et al*.* (2010) was calculated in an Excel sheet and the majority consensus NJ tree with 100 bootstraps calculated. Bootstrap values higher than 80 % are indicated. **c** The corresponding NJ tree for individual accessions is shown

Multifactorial analysis (MFA) in DARwin5 (Perrier et al. 2003) involved calculating simple match scores for all pairs of markers, recorded as fractions of shared markers (Jing et al. 2010). The NJ tree (Fig. 1) was calculated from allele frequencies within the population sub-groups of Jing et al. (2010) in an Excel sheet. Correspondence analysis (Supplementary Figure 2) was performed using DARwin5 (Perrier et al. 2003) to calculate Dice genetic distances between pairs of accessions for the 45 or 27 genetic marker data sets.

### Geographical relationships

Latitude and longitude data were plotted in Google Earth using .kml files (Supplementary Tables 2a and 2b) with the image simplified using Photoshop CS3. Great circle distances were calculated from 35°47′N 72°36′E using the relationship: *y*_i_ = 2 arcsin√[sin^2^(*a*/2) + cos(θ_c_)cos(θ_i_)sin^2^(*b*/2)]. The values *a* and *b* are given as: *a* = θ_c_ − θ_i_ and *b* = φ_c_ − φ_i_; all angles were in radians, where θ and φ are latitude and longitude, θ_c_ is the latitude of the reference point and θ_i_ is the latitude of the sample.

### Data storage and visualisation

The original RBIP data of 45 RBIP markers derived from Jing et al. 2010 are stored in the Germinate Pea database http://bioinf.scri.ac.uk/germinate_pea/app/. These include the 27 markers used in this study. Corresponding data for the 1,518 extra lines scored with these 27 RBIP markers have been stored in the same database.

### Selection of a representative set of accessions

Accessions with high values of *Q* corresponding to the STRUCTURE sub-groups of Jing et al. (2010) and the STRUCTURE groups and sub-groups from the analyses presented here were identified together with outlying accessions in the MFA; several accessions were identified in both procedures (Supplementary Table 1).

Core Hunter software (Thachuk et al. 2009) was used to select accessions for core collections using a multiobjective measure, which consisted of an equally weighted contribution of Modified Rogers’ distance, Cavalli-Sforza and Edwards distance and Shannon–Weaver index, Core sub-sets for seven accessions (the minimum core), and 5, 10, 20 and 30 % of the full set of accessions were derived (Supplementary Table 1). To investigate the propensity of accessions to be sampled by Core Hunter, the 30 % selection was resampled to yield an alternative 10 % core. The overlap between the two 10 % subsets is 280/453, when 151 would be expected by chance alone (Table 2), indicating that Core Hunter shows a tendency to resample the same accessions.

**Table 2:** Statistics of inclusion rates of accessions in different representative samples

Sample	S	CH5	CH10	CH20	CH30	Resample
Number	141	226	453	906	1,359	453
		CH5	CH10	CH20	CH30	Resample
S vs.:	Observed	43	63	81	84	59
	Expected	7.01	14.05	28.09	42.14	47.00
	χ^2^	184.84	170.59	99.63	41.58	3.06
			CH10	CH20	CH30	Resample
CH5 vs.:	Observed		193	197	187	177
	Expected		22.52	45.03	67.55	75.33
	χ^2^		1,290.89	512.86	211.25	137.21
				CH20	CH30	Resample
CH10 vs.:	Observed			336	339	280
	Expected			90.26	135.39	151.00
	χ^2^			669.03	306.19	110.21
					CH30	Resample
CH20 vs.:	Observed				534	331
	Expected				270.78	302.00
	χ^2^				255.86	2.78

The samples compared are coded as follows: S the 141 accessions selected on the basis of STRUCTURE groups and MFA outliers, CH5, CH10, CH20 and CH30 are the sets selected by Core Hunter as representing 5, 10, 20 and 30 % of the accessions, respectively. The column headed ‘Resample’ corresponds to 453 accessions sampled by Core Hunter from the 1,359 in CH30. The row observed is the number of accessions occurring in both samples as on the left and in the column header. The row expected is the number expected on the basis of the relative sample sizes and total number of accessions. The row χ^2^ is the Chi-squared value of the contingency test of the number observed versus expected

The average of the observed/expected ratio for the three combinations for each sample is: S, 3.87; CH5, 5.46; CH10, 4.82; CH20, 3.24; CH30, 2.31; and for resampled, 1.64, suggesting that, normalised for the number of accessions sampled, the accessions sampled by Core Hunter at 5 % representation are those most likely to be resampled by another method

## Results

### Partitioning *Pisum* accessions into groups

The analysis of Jing et al. (2010) partitioned 3,020 accessions of the John Innes (JI) *Pisum* collection into a hierarchical organisation comprising, three main groups which subdivided into 14 sub-groups, some of which were more clearly distinct than others. To test these assignments, a set of 37 *P. sativum* accessions that were strongly assigned to these STRUCTURE sub-groups (Fig. 1a) was selected for more detailed marker analysis. These were screened using 1,484 *PDR1* SSAP markers (Ellis et al. 1998; Knox et al. 2009) of which 625 amplicons were found to be polymorphic in this data set. From this information, neighbour joining trees were constructed for the sub-groups and accessions (Fig. 1b, c) using the DARwin5 software (Perrier et al. 2003).

### RBIP analysis of *Pisum* from European germplasm collections

A new sample set was assembled from major European *Pisum* germplasm collections, comprising 422 accessions from France, 368 accessions from Spain, 295 accessions from Russia, 212 accessions from Poland, 171 accessions from The Netherlands and 50 accessions from Portugal (Supplementary Table 1). DNAs from these samples were scored for 27 RBIP markers that were a subset of those 45 previously investigated (Jing et al. 2010). The resulting marker data were combined with the corresponding existing data for the same markers in the JI *Pisum* collection to produce a sample set of 4,547 containing 9 replicated samples. RBIP markers score the presence and absence of an insertion site simultaneously, however, that does not necessarily signify heterozygosity because either sequence may be duplicated. Here, and previously, such marker states are treated as missing data as discussed in Jing et al. (2010). In total, the allele calls were 42,339 occupied site alleles, 54,956 empty site alleles and 25,474 data points treated as missing data. This is a slightly lower frequency of missing data points than in the previous data set (χ^2^ = 83). The frequency distribution of missing marker scores in the data set is shown in Supplementary Figure 3.

### Correspondence between data sets

To test correspondence between the old and new data sets, two measures of pairwise genetic distance were made for 394 *Pisum* accessions previously scored, one (D_45_) with the set of 45 markers and the other (D_27_) with the set of 27 markers. These two values were strongly correlated (*r* = 0.9, Supplementary Figure 2).

### Bayesian analysis of population structure

The program STRUCTURE (Pritchard et al. 2000; Pritchard and Wen 2004) has been widely used for the description of genetic variation, and analysis with this method formed the basis for the general conclusions drawn by Jing et al. (2010). Here, we undertook the same analysis with the 4,547 samples scored for 27 selected markers. As before (Jing et al. 2010), there was no strong indication of the most appropriate value of *K*, the number of proposed ancestral populations. The value *K* = 2 partitioned the data set robustly, roughly separating group 2 of Jing et al. (2010) from the rest of the germplasm (data not shown), but failed to resolve the most distinct germplasm set, namely group 3, which contains the large majority of wild and primitive cultivated *Pisum* germplasm of the JI *Pisum* germplasm collection.

At *K* = 3, the correlation between the output of 20 independent STRUCTURE runs fell into 6 classes, according to a hierarchical cluster analysis of the inter-run correlations (Supplementary Figure 1a). Three of these classes, comprising half of the runs were more closely related to each other than the others and these were taken for further analysis (Supplementary Figure 1b, c).

**Fig. 2:**
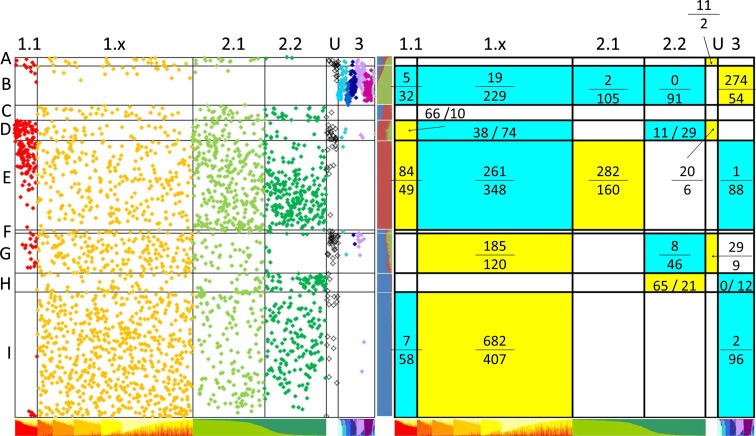
Comparison of STRUCTURE analyses. The *bottom horizontal ribbon* (for *both panels*) shows the assignment to STRUCTURE sub-groups by Jing et al*.* (2010). The *central vertical ribbon* shows the assignment of *Q* values with the data set from this study (see Supplementary Figure 1c). The *upper green* group is referred to as *Q*_G_ and the *red-brown* and *blue* groups as *Q*_R_ and *Q*_B_, respectively. *Spots* in the *left panel graph* indicate the locations of accessions in the two analyses. Observed versus expected numbers are indicated as a fraction for combinations of cells in the *right panel*, which are significantly different from expectation on the basis of a contingency test. Cells highlighted in *yellow* have significantly more accessions than expected and those highlighted in *turquoise* have fewer than expected

The assignment of parentage to those accessions common to this study and Jing et al. (2010) was compared between the two data sets (Fig. 2). The overall correlations between the assignments of *Q* for the corresponding groups are: *Q*_G1_, *Q*_B_ 0.32, *Q*_G2_, *Q*_R_ 0.34 and *Q*_G3_, *Q*_G_ 0.95, where G1, G2 and G3 are the main STRUCTURE groups of Jing et al. (2010) and *Q*_B_, *Q*_R_ and *Q*_G_ refer to the STRUCTURE groups of the extended data set (Supplementary Figure 1b). There were also weaker but significant correspondences between the two other groups (*Q*_B_ and *Q*_R_) and sub-groups of these analyses (Fig. 2). Sub-group 1.1 of Jing et al. (2010) (the first sub-group along the *x* axis, coloured red) is noticeably distinct and seems to correspond mainly to the components of *Q*_R_ in the new analysis and for high assignments to *Q*_R_ (Fig. 2).

**Fig. 3:**
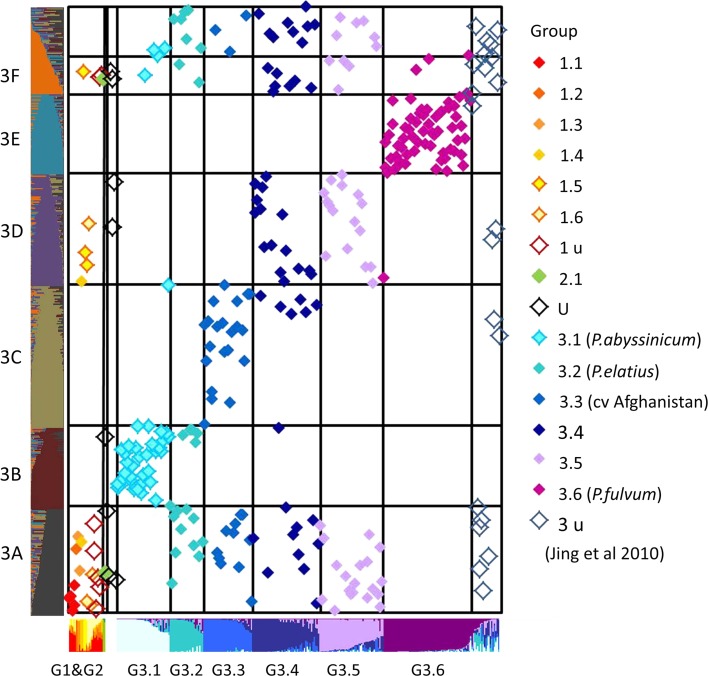
Relationship between STRUCTURE groups containing exotic germplasm. Accessions assigned to the ‘exotic’ group (*Q*_G_) of the *K* = 3 analysis of this data set (the green group of Supplementary Figure 1b) were analysed by STRUCTURE and the most consistent assignment into six sub-groups is presented. The locations of accessions analysed by Jing et al*.* (2010) (*x* axis) are marked by *points* (*x*, *y*) corresponding to their position in that and the analysis of the present data set (*y* axis). The *diamond symbols* at (*x*, *y*) are colour coded according to the scheme of Jing et al. (2010) as indicated on the *right*. Taxonomic groups strongly represented in sub-groups are indicated in *brackets*

The strong correspondence between group 3 and the small group (*Q*_G_), identified in the present analysis, was examined further by repeating the STRUCTURE analysis on this subset of accessions, using the *K* = 6 value derived for group 3 by Jing et al. (2010) [no other *K* value was strongly suggested by STRUCTURE or the method by Evanno et al. (2005)]. 16 out of 20 STRUCTURE runs were very strongly correlated and the average of these is compared to the previous data in Fig. 3. There are three very clear correspondences, namely between the pairs G3.1 and 3B, G3.3 and 3C, and G3.6 and 3E. The accessions previously assigned to sub-groups G3.4 and G3.5 are distributed among sub-groups 3A, 3D and 3F from the new study.

### Multifactorial analysis of *Pisum* population structure

**Fig. 4:**
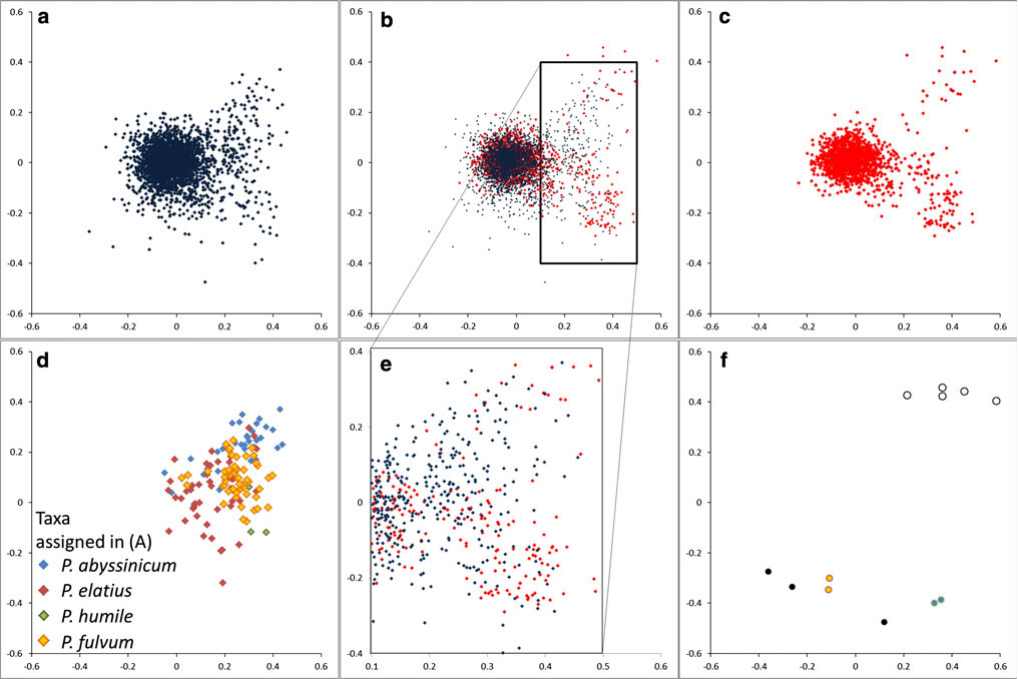
Multifactorial analysis of *Pisum* diversity. Distribution of accessions in MFA: these two dimensions explain 7.24 and 3.61 % of the variance, respectively (note that the variance is distributed over 4,532 dimensions). **a** Accessions previously analysed by Jing et al. (2010) **b** All accessions in the present analysis. **c** Accessions new to this analysis. **d** Accessions in **a** assigned to four main taxa other than *P. sativum*. **e** Region centred near (0.3, 0). **f** Outlying points

A genetic distance matrix was calculated for 4,532 of the 4,547 samples (15 accessions had high numbers of missing scores, such that some pairwise comparisons could not be calculated) and a multifactorial analysis (MFA) performed on it (“Materials and methods”). The results for the first two dimensions of the resulting MFA are shown in Fig. 4. There is a broad overlap in the distribution of the accessions in the new and old data set (Fig. 4a–c), but in the outer region of the plot (boxed in Fig. 4b and highlighted in Fig. 4d, e) there is reciprocal excess or deficiency between the prior and new data. The region near coordinates (0.4, −0.2) is enriched in new accessions (mainly from the Dutch germplasm collection) and some accessions assigned to *Pisum humile.* The region enriched in the old data set (Jing et al. 2010), near coordinates (0.2, 0.1), co-locates with many *P. elatius* and *P. fulvum* accessions (Fig. 4d, e).

A small number of accessions are peripherally located (Fig. 4f). Those in the upper portion of the multifactorial plot correspond to material from the Polish germplasm collection; four are annotated as *P. abyssinicum* (POZP17, POZP18, POZP20, POZP120) and one (POZP12) is assigned to *P. elatius*.

The outlying accessions at the lower part of the MFA (Fig. 4f) are from the old data set (Jing et al. 2010). Three of these (filled grey in the figure) have more that 50 % missing marker data, so their location is probably unreliable. The others have less missing data and are presumably truly diverse at the combination of loci scored here.

### Comparing samples from different germplasm collections

**Fig. 5:**
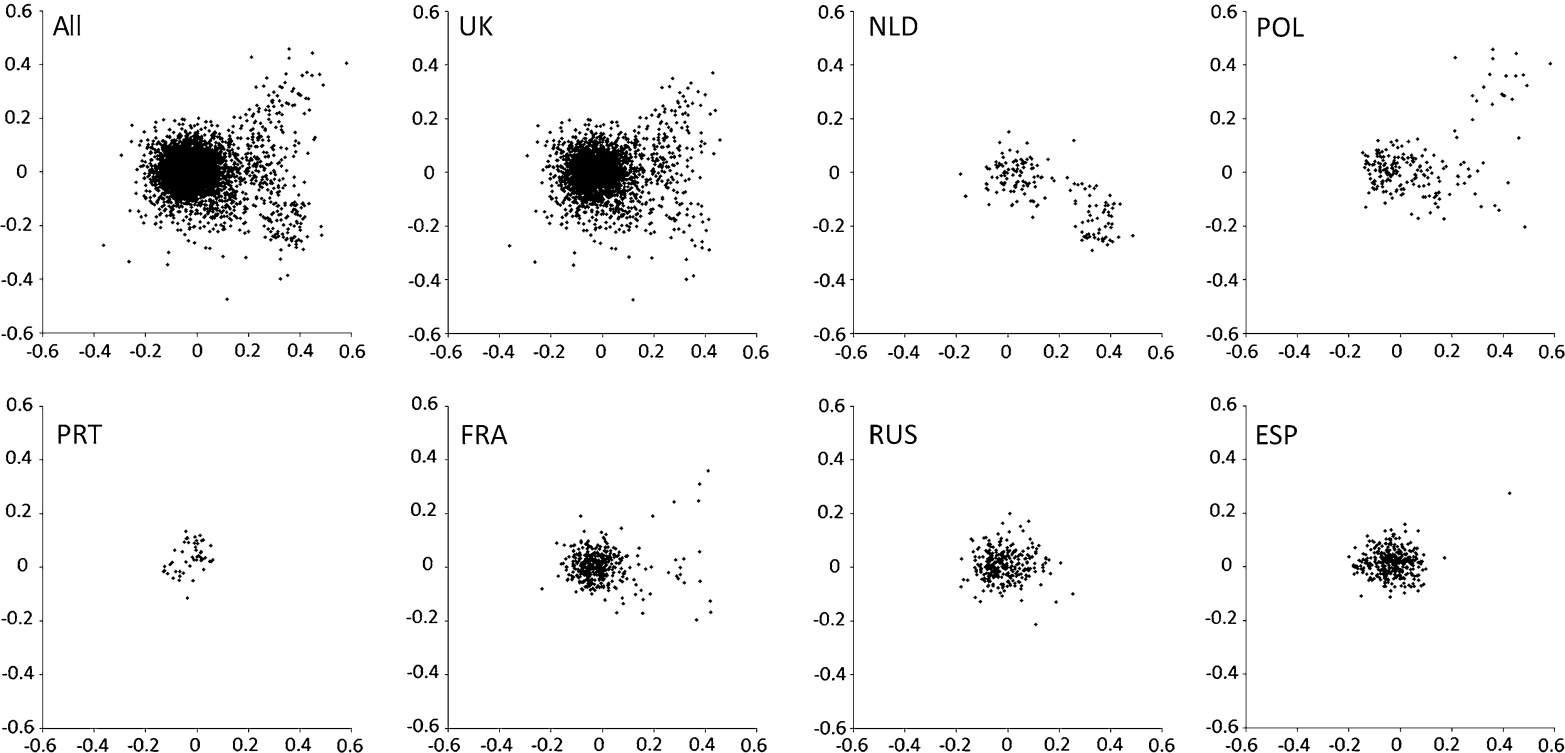
Distribution of accessions by donor. This figure reproduces the MFA plot of Fig. 4, and shows the relative position of accessions obtained from various European germplasm collections. The codes are: *All* the complete data set, *UK* the John Innes *Pisum* collection, *NLD* the Dutch pea collection (Wageningen), *POL* the Polish pea collection (Wiatrowo), *PRT* the Portuguese pea collection (Elvas), *FRA* the French pea collection (Dijon), *RUS* the Russian pea collection (at the Vavilov Institute St. Petersburg), *ESP* the Spanish pea collection (Valladolid)

**Fig. 6:**
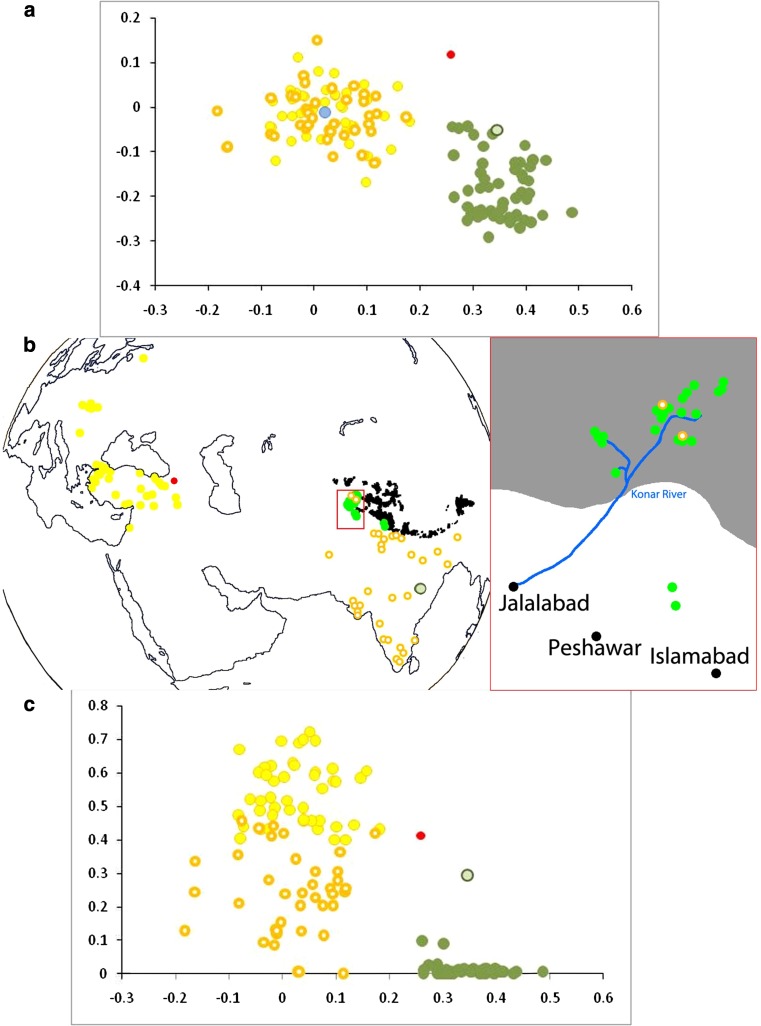
Geographic and genotypic partitioning of Dutch accessions. **a** Multifactorial plot (from Fig. 5 NLD), colour coded. **b** Location of assigned collection points for accessions. The *pale blue spot* in **a** corresponding to a Mexican accession is not shown. The *yellow spots* in the Indian sub-continent are marked as *ringed symbols*. For reference, high points in the Himalayan range are marked in *black*. The cluster of accessions from northern Pakistan is *boxed* in *red* and shown expanded to the *right*, with the mountainous region *shaded grey*. For scale, the distance between Jalalabad and Islamabad is ~250 km. **c** The centre of the group of accessions corresponding to the *solid green spots* tightly clustered in northern Pakistan is at 35°47′N 72°36′E near Mahodand Lake south west of the Karakoram Mountains. The great circle distance from this point (in radians) for all accessions (*y* axis) is plotted against PC1 (*x* axis). Accessions are coloured *yellow* or *green* according to the two main groups in **a**. The *yellow* group is subdivided into *ringed* and *solid colours* according to the location shown in **b**. Three exceptions are: *red* an accession not clearly assigned to either *yellow* or *green*, *blue* a Mexican accession and the *pale green ringed symbol* that corresponds to an accession in the *green* group that was collected at a more southern location (indicated in **b**)

The distribution of accessions by germplasm collection is illustrated in Fig. 5. All of these collections contain much material from the central region of the plot, which includes modern cultivated *P. sativum*, and the peripheral germplasm we have analysed mainly derives from the UK (JIC), Dutch (NLD), French (FRA) and Polish (POL) collections. The Dutch accessions sampled (Fig. 5 NLD) are partitioned into two main genotypic groups and these are explored further in Fig. 6. Collection site information (latitude/longitude coordinates) is available for all of these accessions and these are plotted in Fig. 6b (with the exception of a single Mexican accession identified as a blue spot in Fig. 6a). Almost all of the spots residing in the central region of the MFA plot in Fig. 6a correspond to samples deriving mainly from Europe, Turkey and adjacent Middle East countries (yellow) and India (ringed, orange). In contrast, the green spots derive from germplasm that is mainly from the Himalayan foothills, mostly in northern Pakistan from the Konar River system. There are a few exceptions to these groupings; two of the Konar River accessions belong to ‘Indian’ genetic types and one Indian accession is of the ‘Konar River’ type (Fig. 6b, c).

If the accessions marked in green represented a selection from pea genotypes in the Indian sub-continent, then we would expect a measure of genetic distinctness correlated with the distance. The first component of the multifactorial plot clearly separates these accessions (Fig. 6a, *x* axis), but when this is plotted against distance (Fig. 6c, *y* axis) no clear relationship between genetic and physical distances is seen.

### Identifying a representative subset of *Pisum* accessions for the future study

**Fig. 7:**
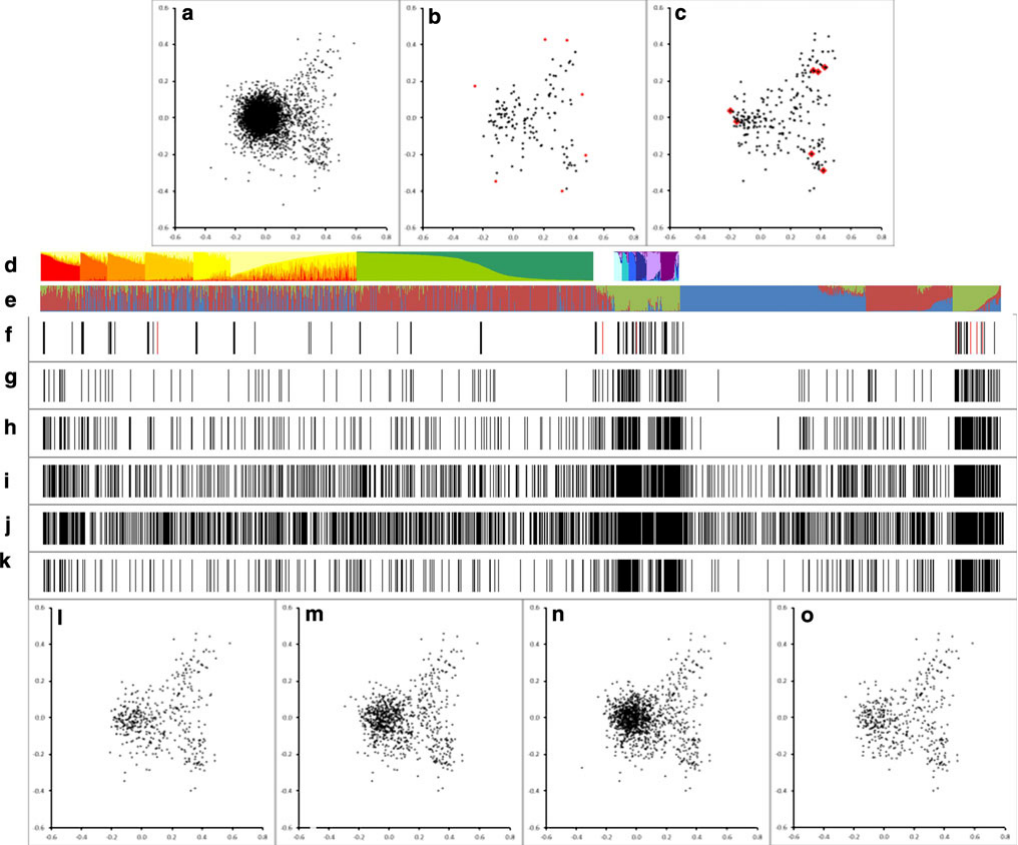
Representative subsets of *Pisum* accessions. Multifactorial analysis (MFA) plots (**a**–**c**, **l**–**o**) and STRUCTURE assignments (**d**–**k**) of selected subsets of accessions are illustrated. The distribution of all accessions in the MFA space is shown in ‘**a**’ (identical to Fig. 4a). The assignments of accessions to STRUCTURE sub-groups of Jing et al. (2010) is shown in ‘**d**’, using their colour codes and accession order. **e** The assignment of accessions to three STRUCTURE groups identified here (*blue* *Q*_B_, *red* *Q*_R_ and *green* *Q*_G_). Those accessions common to **a** and **b** are in the order of Jing et al. (2010), but those on the *right* (new to this analysis) are in the order of Supplementary Figure 1c, with *Q* assigned as the averages of panel A1. **b**, **f** 141 accessions sampled on the basis of STRUCTURE assignments (*black*) and MFA (*red*). **c**, **g** Samples selected by Core Hunter at 5 % representation (*black*) with the seven accessions also in the minimum core highlighted in *red*. The 10, 20 and 30 % Core Hunter selections are in **l**–**n** (MFA) and **h**–**j** (STRUCTURE), respectively. **o**, **k** 10 % representation reselected from the 30 % selection

Two approaches were taken to identify subsets of accessions that represent the genetic diversity present in the germplasm studied here (Fig. 7). The first combined the STRUCTURE and multifactorial analyses in this and our previous studies (Jing et al. 2010). 14 sub-groups of accessions were identified in the previous study and here 3 groups have been identified; the most diverse of which has been further subdivided into 6 sub-groups, together giving 23 groups and sub-groups. Six accessions strongly assigned to each of these 23 groupings were selected by their high corresponding *Q* values (Supplementary Table 1). This should correspond to 138 accessions, but the number was 134 because some individuals were selected from both a group and a sub-group. These were augmented with the 7 outliers in the MFA plot discussed above, to maximise the represented diversity, giving 141 accessions. The distributions of these accessions in the STRUCTURE and MFA plots are shown in Fig. 7b, f.

The second approach for generating representative germplasm core subsets used the Core Hunter program (Thachuk et al. 2009; http://corehunter.org), which identifies subsets of representative accessions on the basis of maximising average genetic distance (“Materials and methods”). Core sub-sets for the minimum core of seven accessions 5, 10, 20 and 30 % of the full set of accessions are shown in Fig. 7 (listed in Supplementary Table 1). To investigate the propensity of particular accessions to be sampled by Core Hunter, the 30 % selection was resampled to yield an alternative 10 % core in which 280 of the 453 were resampled when 151 would be expected by chance alone (Table 2).

The distribution of these sampled accessions with respect to each other within both STRUCTURE and MFA plots is shown in Fig. 7, and the analysis of the frequency with which individual accessions are represented in the different sets is presented in Table 2. The smaller sets (with fewer than 500 members S, CH5 and CH10 in Table 2) selected accessions that were most likely to occur in other selections and the Core Hunter 5 % set performed best by this measure (Table 2). These selections generally over-represent alleles with respect to the data set as a whole; the most extreme is for 1006nr13 (AJ966283). The smaller suggested core samples tend to have the most extreme over-representation of rare alleles and the Core Hunter method has a greater over-representation than the STRUCTURE/MFA-based selection method.

## Discussion

The main purpose of our study was to use molecular markers to determine the range of overlap, and extent of distinctness, of germplasm held in different collections. Our results show that the assignment of accessions to groups and sub-groups as presented by Jing et al. (2010) is broadly corroborated for a smaller set of accessions analysed with 12-fold more markers (Fig. 1). The SSAP markers used for this experiment share the property with RBIPs of being based upon the insertion polymorphism of retrotransposons, but the latter is a codominant single locus approach and the former a dominant multi-locus approach (Waugh et al. 1997; Ellis et al. 1998) similar to transposon display (Van den Broeck et al. 1998). The SSAP analysis broadly supports the conclusions of Jing et al. (2010), notably for the accessions in sub-groups 3.3, 3.4 and 3.5; the close association of the sub-groups 2.1 and 2.2, and the lack of clear subdivision within group 1 except for sub-group 1.1. The similar results from these two marker approaches supports our assertion that the small number of markers used here has yielded reliable genetic diversity data for the 4,532 samples studied using them. Furthermore, the 27 RBIP marker set used reports essentially the same inter-accession distances as for Jing et al. (2010) suggesting that both measures report essentially the same features of genetic distances that distinguish these accessions, further reinforcing our approach. The slightly lower proportion of missing data in the data set we analysed suggests that the new data is at least as robustly scored as the previously analysed data set, even though the number of loci scored is reduced. This is most likely because of technology improvement, in particular the use of a dye swap during the TAM hybridization step for all markers (Jing et al. 2007a).

We have used two main approaches to analyse the marker data, namely STRUCTURE and MFA. The three STRUCTURE groups of accessions studied here correspond approximately to the three groups of Jing et al. (2010) with a strong correspondence between group 3 (coloured blue/purple in Fig. 2a) and the smallest of the three groups identified here, coloured green (*Q*_G_), reflecting the robust assignment of accessions to the ‘wild’ material of STRUCTURE group 3 and the less reliable assignment for the cultivated germplasm which has lower intrinsic genetic diversity. Although the assignment of accessions to sub-groups within STRUCTURE group 1 is not robust (Jing et al. 2010), and was considered poorly resolved, the distinctness of the sub-group 1.1 is again notable (Figs. 2, 3, 7).

Although we tried to maximise the genetic distinctiveness of the new germplasm sampled here, much of the germplasm collected is closely related to cultivated *P. sativum* (Fig. 4). This occupies the central region of the multifactorial plots (Figs. 4, 5) and its dense packing in this region gives the impression that these accessions are highly similar, but this is to some extent misleading. Cultivated *P. sativum* displays lower genetic diversity than wild and primitive cultivated *Pisum*, but the large majority of marker alleles described in the latter can be found in the former (Vershinin et al. 2003). The major difference between these two germplasm classes is the variety of marker combinations (haplotypes) in the wild germplasm.

This study is consistent with the previous analyses and again emphasises the distinctness of *P. sativum* cv. Afghanistan and the taxa *fulvum* and *abyssinicum.* *P. elatius* accessions are concentrated in 3A and 3F, along with accessions previously assigned to G1.1, G3.3, G3.4 and G3.5, consistent with *P. sativum* having been domesticated from this taxon (Vershinin et al. 2003, Jing et al. 2010). These are, therefore, good candidates for extant representatives of *P. sativum* that are most closely related to the wild taxa (Jing et al. 2010).

Two new classes of accessions, deriving from the Dutch (Fig. 6) and Polish collections (Supplementary Figure 4), respectively, have also been identified. In some ways, this is a relatively modest addition to the total genetic diversity available and underscores the breadth of the JI collection, which has captured the majority of the genetic diversity studied here. Nevertheless, this result underlines the need for caution in interpreting the genetic diversity of species on the basis of data from a single collection, even if it is large. It is clear that the JI collection contains the majority of the collected diversity available, but it nevertheless is poorly represented in two sectors of the global diversity of *Pisum* and it is possible that other sectors of *Pisum* diversity remain to be described.

In the Polish collection, the most unusual material corresponds mainly to populations assigned to *P. abyssinicum* or *P. elatius*. These new accessions seem remarkably diverse relative to previous studies which concluded that the genetic diversity of *P. abyssinicum* is strikingly compact (Ellis et al. 1998; Pearce et al. 2000; Vershinin et al. 2003, Baranger et al. 2004; Jing et al. 2005, 2007b, 2010; Smýkal et al. 2011) indicating that these are distinct from ‘*P. abyssinicum*’ as previously used (Supplementary Figure 4). The pattern of relatedness among these accessions suggests three possibilities: (1) these accessions may represent novel genetic variation within *P. abyssinicum*, (2) these accessions may be admixed *P. abyssinicum*, or (3) these accessions may be misclassified. Whichever of these is the case, the accessions are worthy of further analysis and have been included in our representative sets of accessions (Fig. 7; Supplementary Table 1; Supplementary Figure 4). 

The novel Dutch accessions analysed here occupy a region of the MFA plot that contains JI material mainly from Turkey, Afghanistan, Iran and North India. This geographical location was recognised by Vavilov (1992) as a potential secondary centre of diversity for *Pisum*. These accessions are novel genotypes and, therefore, of particular potential interest to breeders and geneticists looking for new sources of variation in this species group. This result also suggests that those accessions held at VIR, sampled by Vavilov and colleagues, but not included in this study would be worthy of detailed molecular marker analysis in the future. The distinctness of these accessions from the Konar River system seems clear, but detailed examination of relatedness and geographical distance is consistent with Indian accessions from nearby showing some degree of admixture with the former that declines with distance from the collecting area (not shown). We conclude that these accessions represent distinct germplasm and note that samples of these are included in all of the approaches we have taken to identify a representative set of accessions (Fig. 7).

There is a lower abundance of accessions of wild species, and the diverse wild and exotic germplasm of group 3 of Jing et al. (2010), in the newly analysed accessions (Fig. 7d) and the corresponding green-coloured STRUCTURE group in Fig. 7e is highly sampled by all the methods used to identify a representative subset of *Pisum* accessions for the future study.

We conclude from these studies that the overall genetic diversity within the genus *Pisum* has been confirmed as a broad continuum with some substructure. Of special note are those accessions of *P. sativum* that are markedly distinct from cultivated types. cv. Afghanistan was recognised as an ‘ecotype’ by Young and Matthews (1982) and it is the source of the *sym2* allele that confers specificity on the symbiotic relationship with rhizobia, plus other distinct loci and traits consistent with it having adapted to a distinct environmental niche. However, its distinctness from other *P. sativum* has not been widely appreciated and it is clear from our study that it is almost as distinct as *P. fulvum*. This cv. Afghanistan class corresponds mainly to a single sub-group (G3.3) in our previous analysis and splits into two classes 3A and 3C in the new data (Fig. 3). While this split does not correlate well to polymorphism at the *Sym2* locus (Young and Matthews 1982), it does correspond to polymorphism at several of the loci studied here (1794-2, 281x16, 399x131 and 45x31). Although cv. Afghanistan is genetically coherent, it is also genetically diverse and the *sym2* allele is distributed between at least two sub-types of cv. Afghanistan.

The inclusion of novel genetic material from northern Pakistan (Fig. 6) has changed the genetic composition of the group of accessions that includes G3 of Jing et al. (2010). It is, therefore, not surprising that our STRUCTURE analysis of this *Q*_G_ partitions the *P. sativum* accessions in a slightly different way from G3.3. Nevertheless, the other two sub-groups of *P. sativum* accessions (G3.4 and G3.5) identified to be most distinct from cultivated types by Jing et al. (2010) have again been identified here, although they show a tendency to occur together (Fig. 3, sub-group 3D).

These data have been used to identify accessions with the potential of representing most of the genetic diversity in European *Pisum* germplasm collections in a far smaller number of accessions (Fig. 7; Supplementary Table 1). No two uses of such selections will necessarily have the same imperatives or requirements, so we have confined ourselves to using broad genetic diversity (assessed by two different approaches) to suggest several core collections of different sizes from seven individual accessions to more than a thousand. A recent search for novel *a* alleles (Hellens et al. 2010) based on the STRUCTURE groups of Jing et al. (2010) identified a rare allele, and this accession was found in all of our core selections, apart from the 5 and 10 % Core Hunter selections, suggesting that our sets of accessions can provide useful genetic diversity.

These selections generally over-represent rare alleles, and a tendency to equalise allele frequencies would be expected for methods sampling distinct haplotypes equally. The Core Hunter samples had the strongest normalisation of allele frequencies and this is the most extreme in the smaller samples. Core Hunter shows a tendency to resample the same accessions. Thus, we are confident that we have characterised the large majority of extant genetic diversity, which is held *Pisum* in germplasm collections and identified genetic variation not previously characterised, with corresponding geographical information on the locations of sources of this additional genetic novelty.

We have used a modest number (27) of markers for the analysis of a relatively large number of accessions (4,538) and compared that to the analysis of smaller subsets (3,020 and 37) with larger numbers of markers (45 and 1,484) noting that the relationships between accessions were broadly similar. The 45 marker set was noticeably easier to analyse than the 27 marker data set suggesting that something of the order of 50 markers would be sufficient for the analysis of ca. 5000 accessions. Analysis of the data set with the complementary model building (STRUCTURE) and analytical (MFA) approaches was helpful in attracting attention to specific subsets of the data. The marker type we employed (retrotransposon insertion sites) is useful because the insertions are not reversible and occur at a relatively low rate (ca. 5 × 10^−7^ per generation, Jing et al. 2005). This marker method is not widely used but neutral SNPs (although reversible) having a mutation rate of the order of 10^−9^ per generation should, in contrast to more rapidly evolving sites, have a low homoplasy rate and be suitable for extensive germplasm surveys.

## Electronic supplementary material

Below is the link to the electronic supplementary material.

Supplementary material 1 (DOC 452 kb)

Supplementary material 2 (XLS 2276 kb)

Supplementary material 3 (KML 10 kb)

Supplementary material 4 (KML 6 kb)
