# Supplementary material for: Genetic diversity in European Pisum germplasm collections
Source: Theor Appl Genet. 2012 Apr 1;125(2):367–80. doi: 10.1007/s00122-012-1839-1 (PMC3385700; doi:10.1007/s00122-012-1839-1)
Supplement: Supplementary file 1 — Supplementary material 1 (DOC 452 kb) [file 122_2012_1839_MOESM1_ESM.doc]

**Supplementary Figure 1. Assignment of STRUCTURE Groups.**

STRUCTURE v2.1 runs were performed with default parameters using K=3 and a 'burn-in' of 10,000 simulations followed by 10,000 MCMC runs.

***Supplementary Figure 1a***


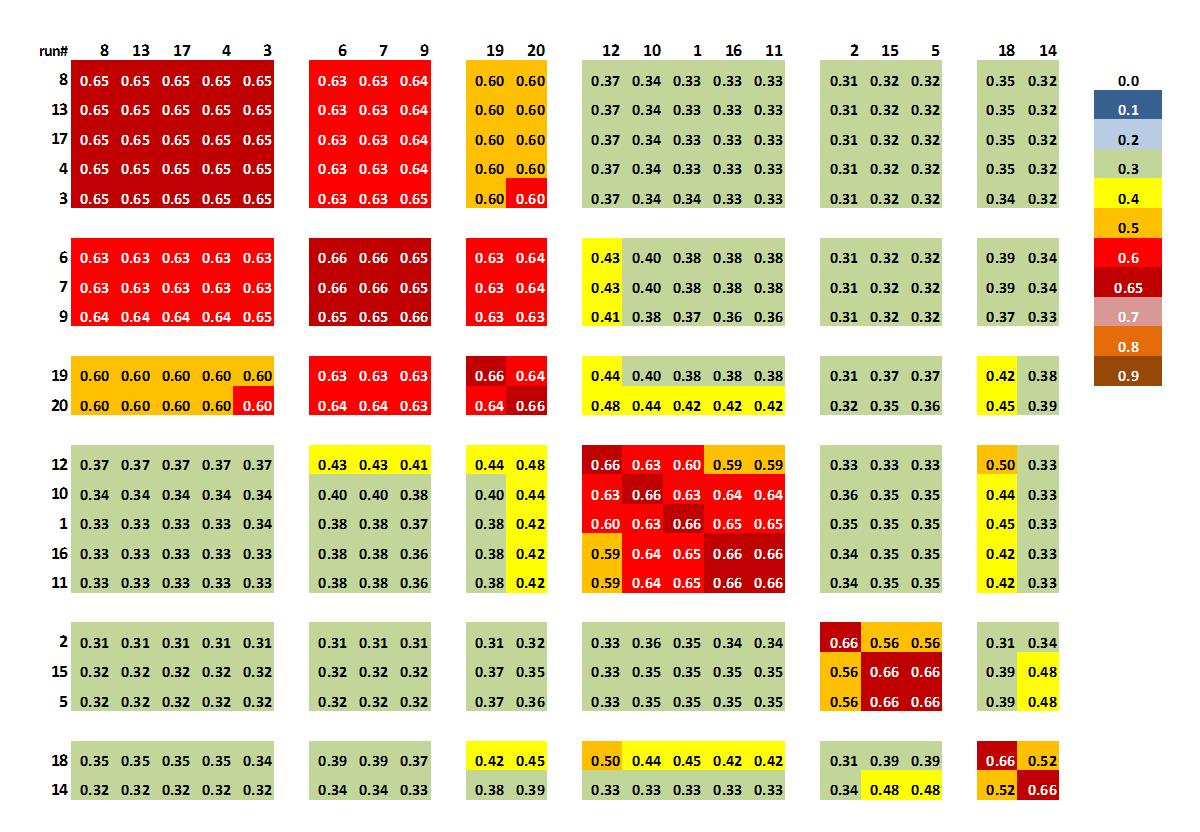


1a: Correlations between 20 Structure runs (see Materials and Methods) are tabulated, separated into groups on the basis of hierarchical cluster analysis in Genstat v13 and coloured according to their value.

***Supplementary Figure 1b***


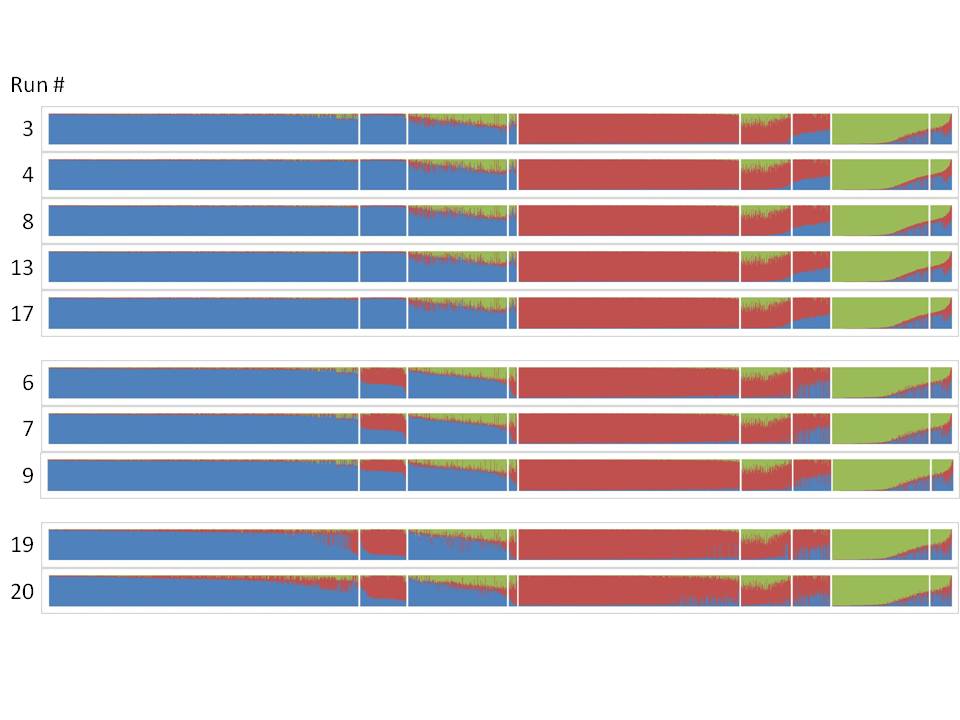


1b: The assignment of Q to the populations Blue (Q_B_), Red (Q_R_) and Green (Q_G_) for the 10 best correlated runs (see 1a) is shown

***Supplementary Figure 1c***


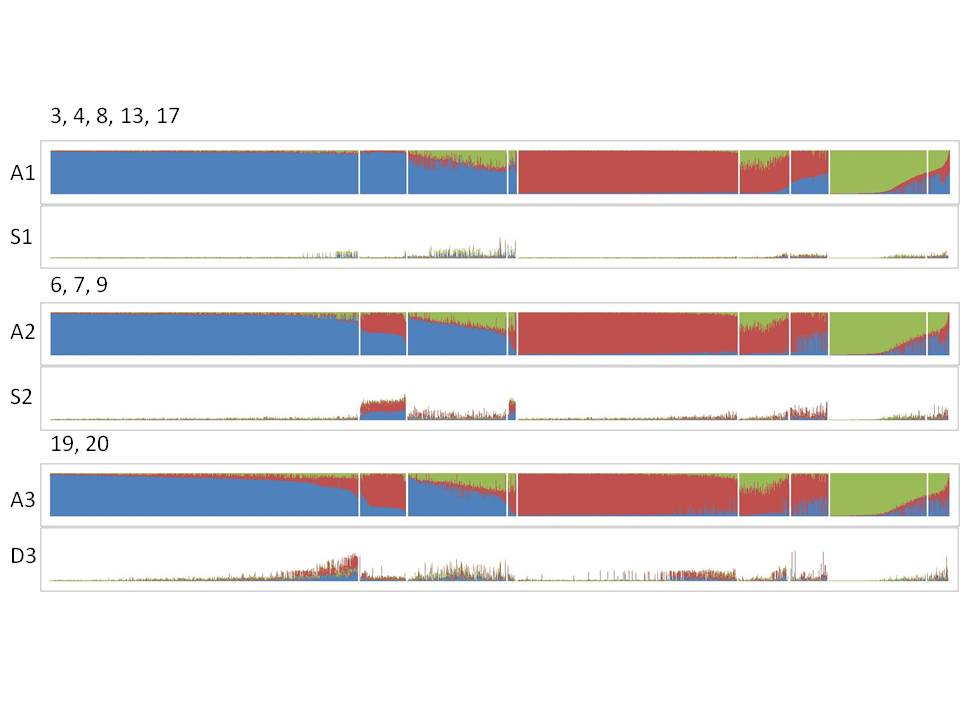


1c: The values of Q_B_, Q_R_ and Q_G_ are averaged for the runs indicated above the panels using groups of runs indicated in 1A. Accession order is the same in 1B and 1C. A1, A2 and A3 are averaged Q values for the runs (3, 4, 8, 13, 17), (6,7,9) and (19,20) respectively. S1 and S2 are the standard deviations corresponding to A1 and A2. D3 is the absolute value of the difference between runs 19 and 20.

**Supplementary Figure 2. Correlation of genetic distance measures**

**
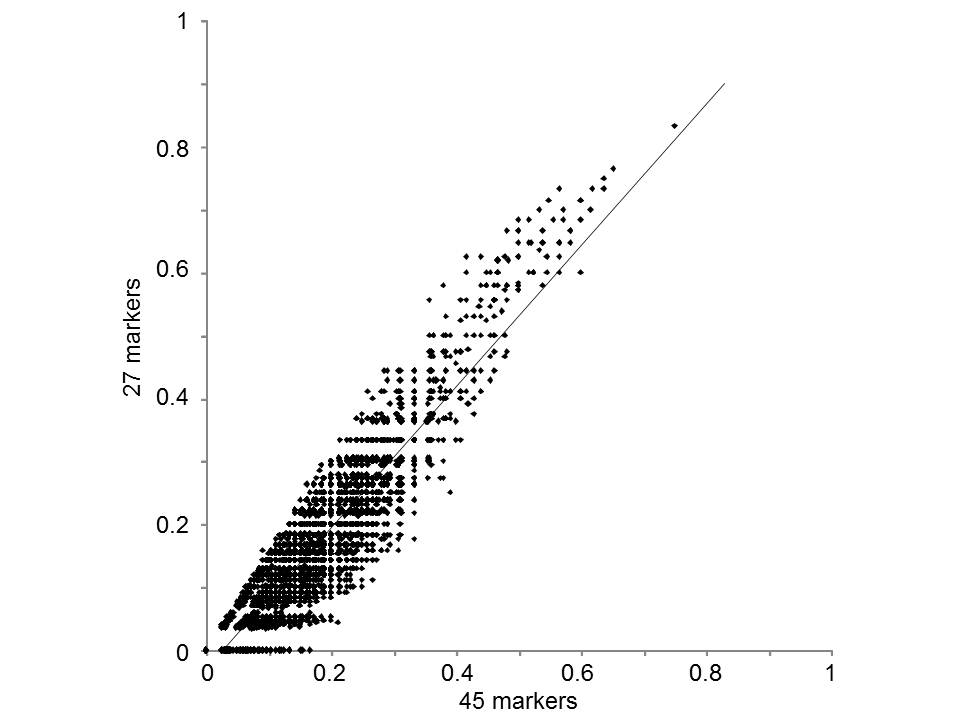
**

394 accessions scored for at least 25 of the 27 markers were selected for this study. For these all pair wise Dice distances were calculated using the program DARwin5 (Perrier *et al.* 2003). Two measures of distance were made, D_27_ and D_45_ as discussed in the text. The data have a correlation coefficient r = 0.9, and followed the relationship D_27_ = 1.12(D_45_) - 0.03 as calculated by Genstat v11 (Payne *et al.* 2008).

**Supplementary Figure 3. Frequency distribution of missing data.**


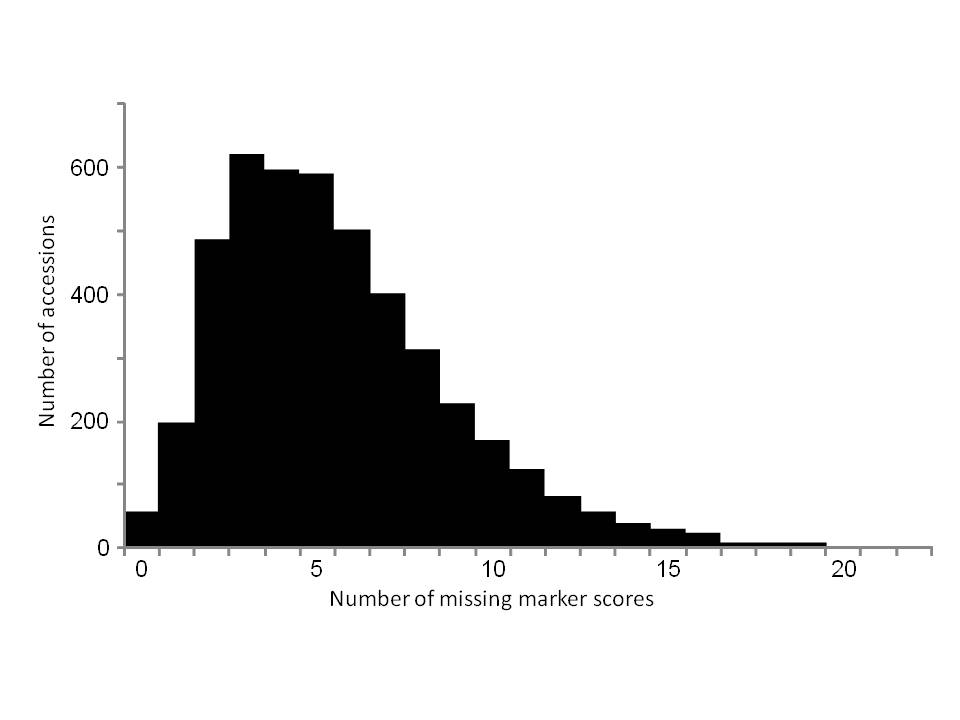


The number of missing data scores (x) is plotted against the number of accessions (y) with that number of missing scores.

**Supplementary Figure 4. Unusual Polish accessions.**


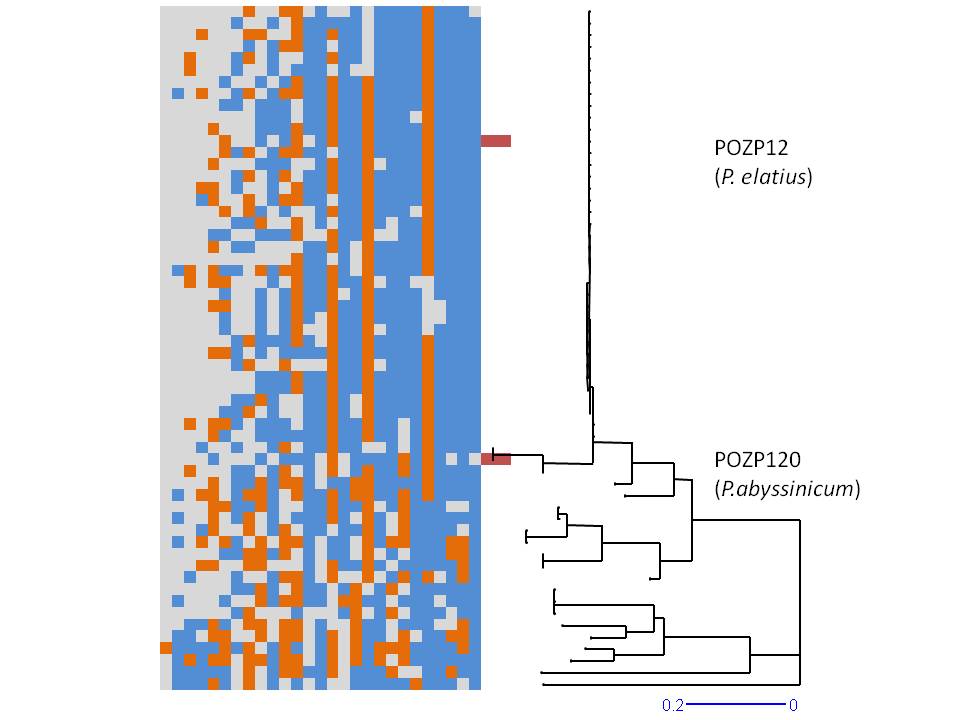


Marker scores for all 27 RBIPs in the *P.abyssinicum* accessions together with POZP12 (upper red mark) and POZP120 (lower red mark) are shown. Genotype scores are coloured blue for empty sites, orange for occupied sites and gray for missing data. The NJ tree to the right was constructed in DARwin5 with a subset of 9 markers selected because they had no missing data for either POZP12 or POZP120. One *P.abyssinicum* accession is missing from the figure as it had 15 missing scores.

The markers (left to right) are: BIRTE-x34, 95x19, MKRBIP2, 281x5, 281x1, 1074CycL29, 1006nr27, 95x2, 2201CYCL6, 2055nr23, 1794x7, 281X44, UniTpv, 1794-1, 1074CycL12, 281x16, Birte-B1, 2385x64, 399_80_46, Birtex16, 399x131, 95x25, 1006nr13, 95x43, 2055nr1, 45x31, 1794-2. The nine used for the tree construction are underlined.

The accessions are (top to bottom)

Source Reference Annotation

JIC 1938 *P. abyssinicum*

JIC 867 *P. abyssinicum* TETRA.

JIC 1632 *P. abyssinicum*

JIC 1869 *P. abyssinicum*

JIC 1458 *P. abyssinicum*

JIC 1961 *P. abyssinicum*

POL027 POZP22 *P. abyssinicum*

JIC 1633 *P. abyssinicum*

POL027 POZP27 *P. abyssinicum*

POL027 POZP5 *P. abyssinicum* POPn - 5

POL027 POZP16 POPULATION - 16

POL027 POZP12 POPULATION - 12 Marked red in figure

JIC 1955 *P. abyssinicum*

POL027 POZP26 *P. abyssinicum*

JIC 1556 *P. abyssinicum*

JIC 1457 *P. abyssinicum*

JIC 1641 *P. abyssinicum*

POL027 POZP24 *P. abyssinicum*

JIC 866 *P. abyssinicum* TETRA.

POL027 POZP17 *P. abyssinicum*

JIC 226 *P. abyssinicum*

JIC 1640 *P. abyssinicum*

POL027 POZP25 *P. abyssinicum*

POL027 POZP1 *P. abyssinicum* POPn. - 1

POL027 POZP21 *P. abyssinicum*

POL027 POZP19 *P. abyssinicum*  BRAUN

POL027 POZP20 *P. abyssinicum*

POL027 POZP18 *P. abyssinicum*

JIC 227 *P. abyssinicum*

FRA043 CRB204 *P. abyssinicum* VAV

JIC 1957 *P. abyssinicum*

POL027 POZP3 *P. abyssinicum* POPn - 3

POL027 POZP10 POPULATION - 10

JIC 2675 *P. abyssinicum*

JIC 130 *P. abyssinicum*

POL027 POZP6 POPULATION - 6

POL027 POZP4 *P. abyssinicum* POPn - 4

JIC 1974 *P. abyssinicum*

POL027 POZP120 POPULATION - 18 Marked red in figure

JIC 225 *P. abyssinicum*

JIC 1943 *P. abyssinicum*

JIC 3046 *P. abyssinicum*

JIC 1998 *P. abyssinicum*

JIC 1876 *P. abyssinicum*

JIC 1966 *P. abyssinicum*

JIC 1937 *P. abyssinicum*

JIC 2079 *P. abyssinicum*

JIC 2006 *P. abyssinicum*

JIC 2 *P. abyssinicum*

JIC 2080 *P. abyssinicum*

POL027 POZP23 *P. abyssinicum*

JIC 2202 *P. abyssinicum*

POL027 POZP11 POPULATION - 11

POL027 POZP13 POPULATION - 13

POL027 POZP3102 OLIWKOWA * ((KOS. * P. ABYS.) * KOS.)

POL027 POZP9 POPULATION - 9

POL027 POZP8 POPULATION - 8

POL027 POZP15 POPULATION - 15

The accession not included was the *P. abyssinicum* accession JI 2674 which had a position remote from most *P. abyssinicum* accessions (Vershinin *et al.* 2003) and is likely of hybrid origin.

**Supplementary Table 1:**

The columns in the excel file Supplementary Table 1 (122_2012_1839_MOESM2_ESM.xls) are:

**Institution Code** These identify the Germplasm collection. Note that the accessions from the JI Germplasm collection are referred to as ‘Jing *et al.* (2010)’ as the samples used in that study are maintained independently as selfed seed progeny. The corresponding JI accession numbers are given in the column ‘Accession Number’.

**Unique Identifier** This is a number that identifies the sample that has been genotyped.

**Accession Number** The number of the accession corresponding to the single plant sample that was genotyped

**NAME / Comment** The name or other information relating to the accession

**Spot Number** The identity of the spot on the array used for genotyping

The columns marked: **281X44, 1794-1, Birte-B1, 2201CYCL6, 399_80_46, Birtex16, 1794-2, 2055nr1, 281x5, 1074CycL29, BIRTE-x34, 2055nr23, 1074CycL12, UniTpv, 281x16, 1006nr27, 95x2, 95x19, 95x25, 1006nr13, 399x131, 95x43, 45x31, 281x1, 2385x64, MKRBIP2** and **1794x7** contain the genotype score for the marker (column) and accession (row) where 1 corresponds to occupied site, 0 to empty site, and -9 to missing data. The missing data are not further classified into sub-type: such as low signal, both present and absent as these have different interpretations for different spots as discussed in Jing et al. (2010). The full data set is available at <http://bioinf.scri.ac.uk/germinate_pea/app/>

The columns: **Axis 1, Axis 2, Axis 3, Axis 4** and **Axis 5** record the position of the accession in the MFA from DARwin5.

**Order in STRUCTURE Q plot** records the sequence of accessions in Supplementary Figure 1B and 1C, and the columns **QB, QR,** and **QG** record the assignments of presumed parentage for the populations Q_B_, Q_R_, and Q_G_.

The columns **3A, 3B, 3C, 3D, 3E** and **3F** record the assignments of presumed parentage to the six populations in Figure 3.

The columns 1.1, 1.2, 1.3, 1.4, 1.5, 1.6, 2.1, 2.2, 3.1 (*P.abyssinicum*), 3.2 (*P.elatius*), 3.3 (cv Afghanistan), 3.4 (*P.sativum*), 3.5 (*P.sativum*), 3.6 (*P.fulvum*) record the assignments of presumed parentage to the fourteen sub- populations of Jing *et al.* 2010 as used in Figure 7. The designations in brackets refer to major component taxa and should not be taken to assign this taxon to all members of that Sub-Group.

The column **representative selection** records ‘1’ the 141 accessions selected on the basis of STRUCTURE group assignments and the multifactorial data.

The column **seven** records (as ‘1’) the accessions identified as the minimum set by Core Hunter (Thachuk *et al. (*2009); http://corehunter.org)

The columns **5% Core Hunter Selection, 10% Core Hunter Selection, 20% Core Hunter Selection, 30% Core Hunter Selection** and **resampled from 30%** record accessions identified by indicated as '1'.

**Supplementary Table 2:**

The files 122_2012_1839_MOESM4_ESM.kml (green spots) and 122_2012_1839_MOESM3_ESM.kml record the data necessary to display the location of the Dutch accessions referred to in Figure 6 using GoogleEarth.
